# Incidence and Risk Assessment of Capsular Contracture in Breast Cancer Patients following Post-Mastectomy Radiotherapy and Implant-Based Reconstruction

**DOI:** 10.3390/cancers16020265

**Published:** 2024-01-07

**Authors:** Maria Vinsensia, Riccarda Schaub, Eva Meixner, Philipp Hoegen, Nathalie Arians, Tobias Forster, Line Hoeltgen, Clara Köhler, Kristin Uzun-Lang, Vania Batista, Laila König, Oliver Zivanovic, Andre Hennigs, Michael Golatta, Jörg Heil, Jürgen Debus, Juliane Hörner-Rieber

**Affiliations:** 1 Department of Radiation Oncology, Heidelberg University Hospital, 69120 Heidelberg, Germany; 2 Heidelberg Institute of Radiation Oncology (HIRO), 69120 Heidelberg, Germany; 3 National Center for Tumor Diseases (NCT), 69120 Heidelberg, Germany; 4 Clinical Cooperation Unit Radiation Oncology, German Cancer Research Center (DKFZ), 69120 Heidelberg, Germany; 5 Department of Gynecology and Obstetrics, Heidelberg University Hospital, 69120 Heidelberg, Germany; 6 Brustzentrum Heidelberg Klinik St. Elisabeth, 69121 Heidelberg, Germany; 7 Department of Radiation Oncology, Heidelberg Ion Beam Therapy Center (HIT), Heidelberg University Hospital, 69120 Heidelberg, Germany; 8 German Cancer Consortium (DKTK), Partner Site Heidelberg, 69120 Heidelberg, Germany

**Keywords:** post-mastectomy radiotherapy, capsular contracture, capsular fibrosis, breast cancer, immediate implant-based breast reconstruction, breast reconstruction

## Abstract

**Simple Summary:**

Capsular contracture is one of the substantial complications of breast cancer patients undergoing post-mastectomy radiotherapy (PMRT) following an immediate implant-based breast reconstruction (IBR). Until now, there has been no therapeutic target to directly treat or manage capsular contracture, which emphasizes the importance of its prevention. A further assessment of risk factors and dosimetric characteristics is needed to minimize the risk and choose the optimal therapy approach.

**Abstract:**

Our study aims to identify the risk factors and dosimetry characteristics associated with capsular contracture. Methods: We retrospectively analyzed 118 women with breast cancer who underwent PMRT following an IBR between 2010 and 2022. Patients were treated with PMRT of 50.0–50.4 Gy in 25–28 fractions. Capsular contracture was categorized according to the Baker Classification for Reconstructed Breasts. Results: After a median follow-up of 22 months, the incidence of clinically relevant capsular contracture (Baker III–IV) was 22.9%. Overall, capsular contracture (Baker I–IV) occurred in 56 patients (47.5%) after a median of 9 months after PMRT. The rate of reconstruction failure/implant loss was 25.4%. In the univariate analysis, postoperative complications (prolonged pain, prolonged wound healing, seroma and swelling) and regional nodal involvement were associated with higher rates of capsular contracture (*p* = 0.017, OR: 2.5, 95% CI: 1.2–5.3 and *p* = 0.031, respectively). None of the analyzed dosimetric factors or the implant position were associated with a higher risk for capsular contracture. Conclusion: Postoperative complications and regional nodal involvement were associated with an increased risk of capsular contracture following breast reconstruction and PMRT, while none of the analyzed dosimetric factors were linked to a higher incidence. Additional studies are needed to identify further potential risk factors.

## 1. Introduction

Immediate implant-based breast reconstruction has been the more preferable therapy approach for breast cancer patients. Compared to a simple mastectomy, breast reconstruction offers better psychological and emotional effects, sexual body image and well-being [1]. In patients with locally advanced breast cancers, post-mastectomy radiotherapy (PMRT) has been demonstrated to improve both local control and breast cancer mortality [2,3]. One of the substantial complications of PMRT following an implant-based breast reconstruction is capsular contracture, which causes pain, swelling and cosmetic failure [4]. Capsular contracture manifests as fibrosis and coarseness, with severe cases including deformation and asymmetric appearance. In these severe cases, a second surgical reconstruction is often needed [2,5].

Radiotherapy is known to be one of the major risk factors of capsular fibrosis. There are various reports regarding the incidence of capsular contracture following PMRT with implant-based reconstruction in the literature, ranging from 16.9% to 67.5% [6,7,8]. A meta-analysis demonstrated that PMRT increased the risk of capsular contracture with a significant odds ratio (OR) of 5.26 (95% CI: 2.73–10.13, *p* < 0.00001), leading to an increased rate of reconstruction failure (OR: 2.59, 95% CI 1.46–4.62; *p* = 0.001) [2].

The pathogenesis of capsular contracture is known to be complex and is not yet completely understood. It is a multifactorial process, postulated to occur through increased inflammation caused by endogenous factors (e.g., healing response modification, surgical technique) as well as exogenous factors (e.g., infection and biofilm formation) [9,10]. At the intercellular level, TGF-ß is known to play a fundamental role in the development of excessive fibrosis, including hypertrophic scars and keloids [11], as the expression of TGF-ß1 and TGF-ß2 was significantly higher in explanted periprosthetic breast capsules [12]. It is reported that the fibroblast amount found in capsular contracture specimens rises with the intensity of contracture [13].

Up until now, there has been no therapeutic target to directly treat or manage capsular contracture, which emphasizes the importance of its prevention. Capsular contracture remains a major and highly relevant adverse event of PMRT on reconstructed breasts. In this study, we aimed to assess possible risk factors for capsular contracture, including the dosimetry characteristics of patients who underwent PMRT following an implant-based reconstruction. Further identification of risk factors can help minimize the risk of capsular contracture and may assist health providers in choosing the optimal therapy approach.

## 2. Materials and Methods

### 2.1. Study Design, Data Collection and Follow-Up

We conducted a retrospective review of breast cancer patients who had received immediate implant-based reconstruction followed by PMRT between 2010 and 2022 at Heidelberg University Hospital. The data collection and analysis were approved by the ethics committee of the medical faculty (S-535/2021).

Patient characteristics as well as tumor classifications, histology, chemotherapy (neoadjuvant and adjuvant), surgical techniques, postoperative complications and radiation dosimetry were registered. Postoperative complications included prolonged pain, edema, seroma and prolonged wound healing. Radiotherapy was administered to all patients with CT-based 3D treatment planning, either in deep inspirational breath hold (DIBH) or free breathing (FB). While most patients up until 2015 were treated with 3D conformal radiotherapy, PMRT has been administered mostly with intensity-modulated radiation therapy (IMRT) or volumetric modulated arc therapy (VMAT) from 2016 onwards.

Patients with regional lymph node metastases were treated with locoregional irradiation of the supraclavicular, axillary and/or internal mammary (IM) levels if national guidelines recommended additional regional nodal irradiation at the time of treatment [14,15,16]. All patients received radiation doses of 50.0–50.4 Gy in 25–28 once-daily fractions for a treatment time of 5 to 6 weeks. 

The incidence of capsular contracture, the time of occurrence and other symptoms were registered. The diagnosis of capsular contracture was based on clinical examination or imaging (MRI and/or ultrasound) and classified according to the Modified Baker Classification for Reconstructed Breasts [17]. In detail, Baker I defines capsular contracture only as shown in imaging; the implant is soft but detectable by physical examination or inspection. Baker II shows mild firmness, while Baker III describes moderate contracture with firmness and visible distortion. Painful and severe contractures with marked distortion are classified as Baker IV. According to the mentioned Baker classification, Baker I–III are still be considered a good or acceptable outcome, meanwhile Baker IV is regarded as a poor outcome. A second reconstructive operation caused by capsular contracture was regarded as reconstruction failure/implant loss. Clinical outcomes, including local control (LC), the occurrence of distant metastasis and overall survival (OS), were further assessed.

### 2.2. Statistical Analysis

The primary outcomes include the occurrence of capsular contracture and reconstruction failure. Possible risk factors included menopausal status, tumor stage, previous chemotherapy, hormone therapy and postoperative complications (prolonged pain, seroma, prolonged wound healing) and were analyzed using a chi-square test or the Wilcoxon rank sum test for categorical or continuous data, respectively. The Kaplan–Meier method was used to visualize differences between the assumed risk factors. Patients were censored either at the first diagnosis of capsular contracture, death or at the last follow-up, measured in months starting the last day of radiotherapy. A *p*-value < 0.05 was considered to be significant. Potentially interacting covariates (with *p* < 0.2 in the primary analysis) were tested with a multivariate analysis using the Cox Regression model to calculate the hazard ratios (HRs). All analyses were performed using SPSS (Version 29; Chicago, IL, USA).

## 3. Results

### 3.1. Patient and Radiotherapy Characteristics

A total of 118 consecutive breast cancer patients (median age: 45 years) who had undergone PMRT between 2010 and 2022 were included in this single-center retrospective review. The median follow-up was 22 months (range: 12.6–34.3 months). Most of the patients (82.2%, n = 97) received chemotherapy. Altogether, 17 patients (14.4%) received both neoadjuvant and adjuvant chemotherapy, while 52 patients (44.1%) received neoadjuvant therapy, and 28 patients (23.7%) received adjuvant chemotherapy. Ninety-three patients (78.8%) were treated with additional endocrine therapy. The patient characteristics are shown in Table 1, stratified by the occurrence of capsular fibrosis.

The patients underwent radiotherapy for a median of 10 weeks (IQR: 6.39–26.25 weeks) following immediate breast reconstruction. Eighty-one patients (68.6%) were irradiated using deep inspirational breath hold (DIBH), and thirty-seven patients (31.4%) were irradiated using free breathing. Eleven patients (9.3%) received PMRT of the chest wall only, while seventy-one patients (60.2%) were treated with PMRT including regional nodal irradiation at the supra- and infraclavicular levels with or without the internal mammary level. In 36 patients (30.5%), the axillary levels I and II were additionally included. Most patients (83.9%) were treated with 50.0 Gy in 25 fractions, while 14.4% received 50.4 Gy in 28 fractions. In five patients (4.2%), an additional boost dose was administered to the former tumor bed due to cutaneous or pectoral infiltration. The radiation techniques included volumetric modulated arc therapy (VMAT) in 102 patients (86.4%) and three-dimensional conformal radiotherapy (3D-CRT) in 16 patients (13.5%). The average planning target volume (PTV) was 1205.7 cm^2^ (range: 369–2867 cm^2^). The mean PTV-D98%, PTV-D50%, PTV-D2% and PTV-Dmean were 36.9 Gy, 50.0 Gy, 52.8 Gy and 48.9 Gy, respectively (Table 2).

### 3.2. Development of Capsular Contracture

**Figure 1:**
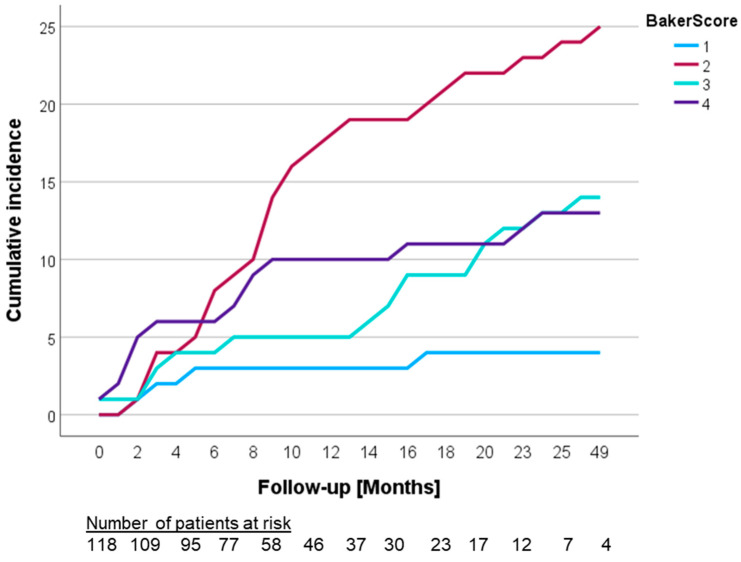
Cumulative incidence based on Baker Classification, applied to the development of capsular fibrosis.

Out of the 118 patients, capsular contracture occurred in 56 patients (47.5%), a median of 9 months after PMRT (IQR 3–16.5 months). Out of the 56 patients with capsular contracture, 4 patients (7.1%) were classified as Baker I, with 25 (44.6%), 14 (25.0%) and 13 (23.2%) patients categorized as Baker II, III and IV, respectively (Figure 1). Across the entire cohort, the rate of Baker III–IV capsular contracture was 22.9%, while Baker IV, classified as a poor outcome, was detected in 11.0%. Among the 56 patients diagnosed with capsular contracture, 30 (53.6%) underwent surgical revision. The overall reconstruction failure rate was 25.4%. Surgical revision included reconstruction with new implants in 5 patients (16.7% of revisions), 14 patients (46.7%) obtained autologous reconstruction and 11 patients (36.7%) underwent implant removal without any reconstruction. Among the five patients who underwent implant exchange, two developed capsular fibrosis in the new implant. Out of the 118 patients who underwent breast reconstruction followed by PMRT, reconstruction failure/implant loss occurred in 25.4%.

### 3.3. Analysis of Potential Risk Factors

**Figure 2:**
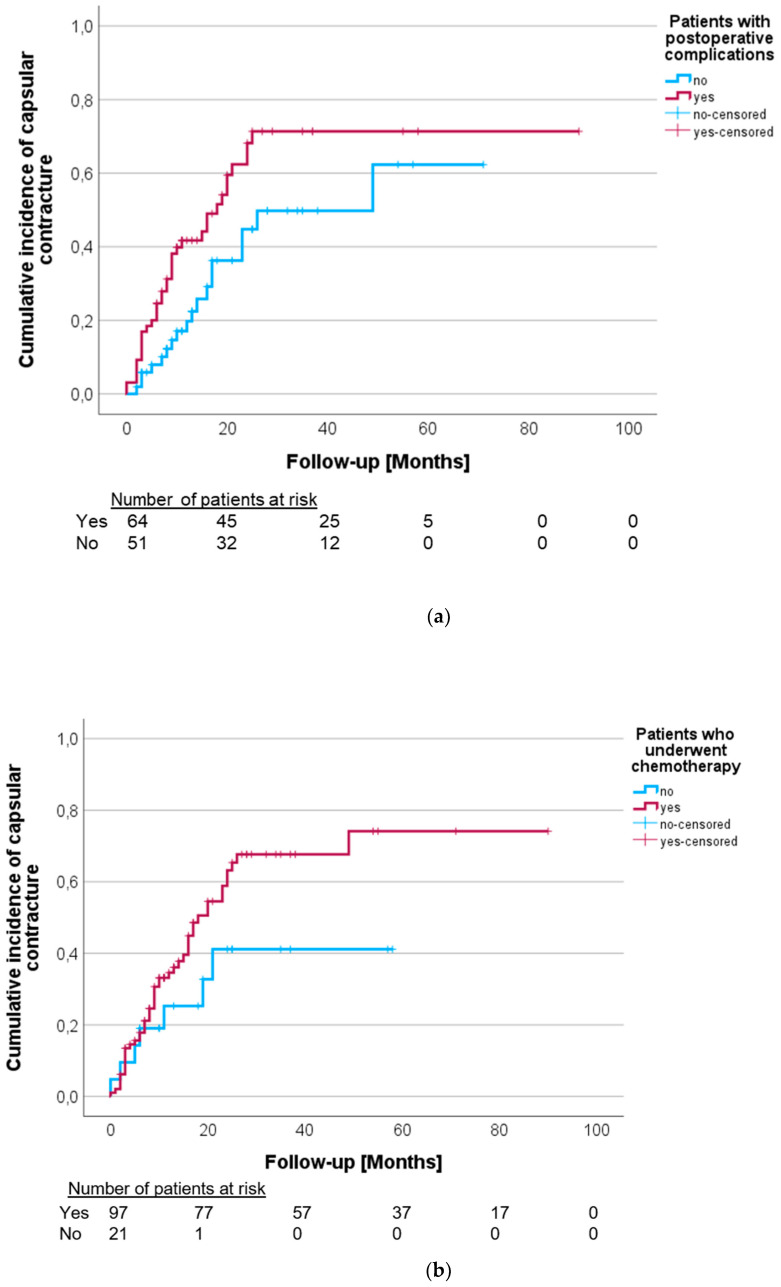
(**a**). Postoperative complications, e.g., prolonged wound healing, prolonged pain and seroma, significantly affect the rate of capsular contracture (*p* = 0.017). (**b**). Cumulative incidence of capsular contracture in patients with and without chemotherapy (*p* = 0.153).

Postoperative complications were significantly associated with a higher risk of capsular contracture (*p* = 0.017, Figure 2a). Postoperative complications prior to PMRT included prolonged wound healing (*p* = 0.065), prolonged pain (*p* = 0.073), seroma (*p* = 0.187) and swelling (*p* = 0.459). Although insignificant, the patients who received chemotherapy might be more susceptible to developing capsular contracture (*p* = 0.153, Figure 2b). Out of the 97 patients who received chemotherapy, 49 (50.5%) developed capsular contracture, while only 7 out of the 21 patients (33.3%) who did not receive chemotherapy developed capsular contracture. Further differentiation between neoadjuvant and adjuvant chemotherapy did not show any significant association, with *p* = 0.223 and *p* = 0.533, respectively. Additionally, the admission of Her2-targeted therapy, e.g., Trastuzumab/Pertuzumab, was not associated with the development of capsular contracture (*p* = 0.769).

Regarding patient characteristics, there were no significant associations between age (*p* = 0.338), menopausal status (*p* = 0.618) and BMI (*p* = 0.834) and the development of capsular contracture. Tumor-related traits, such as histological subtype, tumor stage, grading and hormone receptor status (ER/PR), also showed no direct association (*p* = 0.579, *p* = 0.500, *p* = 0.120 and *p* = 0.247/*p* = 0.149, respectively). Interestingly, we found a significant correlation between nodal status and the incidence of capsular fibrosis (*p* = 0.031). However, lymph node surgery (sentinel node vs. axillary dissection, *p* = 0.744) and surgical technique (nipple sparing mastectomy, skin sparing mastectomy or modified radical mastectomy, *p* = 0.763) were not significantly related to capsular contracture (Table 1). Modified radical mastectomy was defined as the removal of the entire breast tissue without areolar preservation, followed by axillary lymph node dissection. Immediate breast reconstruction was mostly carried out with permanent implants, while six patients received a temporary tissue expander. A total of 106 patients (89.8%) received prepectoral implants, while in 13 patients (11.0%), it was placed beneath the pectoral muscle (subpectoral). There was no significant difference in both groups (*p* = 0.472). Other analyzed implant and surgical characteristics included implant volume, implant surface, its shape and the use of meshes. None of these factors showed any significance (*p* = 0.531, *p* = 0.622, *p* = 0.940 and *p* = 0.074; Table 1).

A detailed dosimetric analysis of radiotherapy (Table 2) did not reveal any significant correlation with the occurrence of capsular contracture. Furthermore, the extent of nodal irradiation was not significantly associated with capsular contracture (*p* = 0.183). Additionally, a larger thoracic wall PTV (mean 1104.1 cm^3^ vs. 989.5 cm^3^, *p* = 0.264) and a larger total PTV (thoracic wall including the nodal irradiation volume) (mean 1255.6 cm^3^ vs. 1185.9 cm^3^, *p* = 0.099) were detected in patients with capsular contracture, albeit not significantly.

In the multivariate analysis, we included pathological nodal status, postoperative complications, perioperative chemotherapy, the extent of the PMRT and total PTV. Here, nodal status and postoperative complications were significantly associated with the occurrence of capsular contracture (*p* = 0.004 and *p* = 0.011, respectively, Table 3). Patients with N2 and N3 were more likely to develop capsular contracture (HR: 2.9, 95% CI: 1.2–7.4 and HR: 6.7, 95% CI: 2.0–22.9, respectively). Out of the 118 patients, 26 unilateral breast cancer patients received bilateral breast implants as reconstruction. Two patients developed capsular contracture on both sides, including the non-irradiated site; meanwhile, one patient developed capsular contracture only on the non-irradiated site. 

During the follow-up, five patients in total (4.3%) were diagnosed with local recurrences, two patients (1.7%) developed contralateral secondary breast cancer and eighteen patients (15.3%) were diagnosed with distant metastases. Seven patients (5.9%) died during the follow-up.

## 4. Discussion

In this retrospective study, we analyzed the prevalence of capsular fibrosis following PMRT and potential risk factors which may favor its development. The rate of capsular contracture of 47.5% detected in the current study is in line with the wide range reported in the literature (16.9–67.5%) [6,7,8]. Capsular contracture often develops later on and increases in frequency even years after reconstruction. Several studies on augmented and reconstructed breast implant patients demonstrated that a longer follow-up period reveals a higher rate of capsular contracture [18,19,20]. Hence, the wide range of incidences reported in the literature is probably influenced by the necessity of a sufficient follow-up duration and the way of performing the assessment, e.g., imaging. With a median follow-up of 22 months, most capsular contractures (24.5%) in this study were asymptomatic and only visible by imaging (Baker I–II). The rate of symptomatic, clinically relevant Baker III–IV capsular contracture was 22.9%. Most patients who received operative revision were those with Baker III–IV capsular contracture, and the overall revision rate was 25%. The revision rate was slightly higher than the rate of clinically relevant capsular contracture, as the individual perception of capsular contracture was highly subjective and therefore could influence the decision for a surgical revision. The incidences of Baker I–II and Baker III–IV were lower than the results reported by Cowen and Gross et al. [6,21], who observed the occurrence of capsular contracture in 141 breast cancer patients who had received implant-based breast reconstruction and PMRT. Cowen and Gross et al. reported an incidence of 67.5% and 32.5% of Baker I–II and Baker III–IV capsular contractures, respectively, with an overall revision rate of 32/141 (22.7%) for a 37-month follow-up.

Another study by Reinders et al. (mean follow-up: 21.75 months) reported an occurrence rate of Baker III–IV capsular fibrosis of 13/80 patients (16.9%) with a 13% rate of surgical revision, which is considerably lower than in our current study [8]. Nava et al. reported in 109 patients (median follow-up: 50 months) a higher rate of capsular contracture of 57.8% without further differentiation regarding Baker classification; however, a considerably lower reconstructive failure rate of only 6.4% was detected [22,23]. It is widely accepted that PMRT following breast reconstruction is associated with an increased risk of secondary breast reconstruction surgery [24]. A large retrospective study on more than 2300 patients demonstrated that patients who received PMRT within 10 months of surgery were significantly more likely to undergo secondary breast reconstruction (57.1%), compared to 36.6% of patients who received no radiation therapy [25]. Another 10-year study on 1788 patients who received implant-based breast augmentation without radiation reported a revision rate of 31.5% [18]. It is important to consider the baseline risk of capsular contracture in the absence of radiotherapy. Hence, the revision rate for patients following PMRT might also include patients who would have had reconstructive failure without the additional application of PMRT. 

Previous studies reported a higher prevalence of capsular contracture in older patients [26]. Most patients observed in this study were <50 years old (74%) and premenopausal (63%). We observed no significant correlation between age or menopausal status and the incidence of capsular contracture. Hammond et al., who analyzed a subgroup of 92 patients undergoing breast reconstruction and PMRT, also did not demonstrate any association between age and the occurrence of capsular contracture [7]. The majority of other reviews on capsular contracture included both patients receiving cosmetic breast augmentation and/or breast reconstruction following mastectomy with or without PMRT [19,26]. This may reflect an age difference between the respective groups: while younger patients received cosmetic breast augmentation, the majority of older patients underwent breast reconstruction and PMRT in lieu of a cancer diagnosis. 

Similar to Cowen and Gross et al. [6], we also observed a significant association between regional nodal involvement (pN/ypN) and the occurrence of capsular contracture (*p* = 0.031). This can probably be explained by the fact that patients with a positive nodal status received a more extensive operation and were therefore more likely to develop postoperative complications, which increased the risk of capsular contracture. Moreover, these patients were more likely to receive chemotherapy and additional lymph node irradiation, which might have also increased the risk. However, the uni- and multivariate analyses did not reveal the extent of lymph node surgery (sentinel node extirpation vs. axilla dissection) or the admission of nodal irradiation to be significantly associated with the development of capsular contracture, most likely due to a rather low number of patients. 

There are various preliminary reports on mouse models about the protective effects of tamoxifen and other hormonal therapies against the development of capsular contracture [27]. Preclinical studies on tamoxifen have shown a decrease in TGF-β1 and TGF-β2 production, which are cytokines that are mostly associated with fibrosis [28]. The transdermal application of tamoxifen to mice was found to be effective in lowering the rate of capsular formation [29]. However, somehow contradictory, various studies also reported a higher risk of capsular contracture in patients with hormone therapy [30]. In this study, the rate of capsular contracture did not differ significantly with the adjuvant use of tamoxifen or other hormonal therapies (*p* = 0.654), which might underline the complex and multifactorial pathogenesis of its occurrence. 

We observed a higher rate of capsular contracture in patients who received chemotherapy. Some studies suggest that the administration of chemotherapy, in addition to radiotherapy, could negatively impact the regeneration of cells and therefore increase the risk of fibrosis and reconstruction failure [31,32] Further differentiation between neoadjuvant and adjuvant chemotherapy did not show any significant effect on capsular contracture in the current study. The protective effect of neoadjuvant chemotherapy, which has been previously reported by Hammond et al., could not be confirmed in this study [7]. However, an extensive risk profiling of breast fibrosis following whole breast radiation therapy with or without boost irradiation reported that concomitant chemotherapy was especially associated with an increased risk of moderate and severe fibrosis [30]. 

In this study, postoperative complications significantly influenced the rates of capsular contracture (*p* = 0.017 in the univariate analysis; OR: 2.488 (95% CI: 1.171–5.287)). These complications included hematoma/seroma, prolonged wound healing, pain and swelling of the surgical site. We found that prolonged pain and wound healing prior to radiotherapy (*p* = 0.073 and *p* = 0.065) were complications, which mostly occurred in patients who later developed capsular contracture. A dysregulation of the normal regenerative response could be further compromised by radiation therapy, as radiation hinders angiogenesis, causing chronic hypoxia and increasing oxidative stress [33]; meanwhile, chronic inflammation increases the production of cytokines and the activation of fibroblasts, resulting in contractures [34]. Studies have shown that contracted capsules possess greater numbers of inflammatory cells such as macrophages, fibroblasts and T-cells [35,36,37], and seroma formation is rich in inflammatory mediators, which might also increase the risk of capsular contracture [4]. 

In this study, most of the reconstructions were carried out using implants, and only six patients (5%) received immediate reconstruction with the application of an expander. Hence, this dataset was too small to observe any effect of the reconstruction type. A meta-analysis from 2017 on 899 cases revealed that the pooled risk of reconstruction failure was not significantly different between PMRT with a tissue expander and a permanent implant [38]. A prospective study from 2016 found no difference in outcomes between PMRT for patients with expanders compared to patients with permanent implants [39]. Nevertheless, two studies reported a higher rate of reconstruction failure after PMRT with an expander compared to a permanent implant [22,40].

Our study showed no significant differences between the surgical methods of a nipple- or skin-sparing mastectomy (*p* = 0.763). There are studies suggesting that, compared to the commonly used prepectoral placement of a breast implant, a subpectoral placement in breast augmentation lowers the risk of capsular fibrosis [41,42,43]. On the other hand, a recent meta-analysis demonstrated that patients undergoing IBR after a mastectomy experienced fewer capsular contractures if the implant placement was prepectoral [44]. In this work, no significant difference was seen regarding implant position (*p* = 0.427), but there was a slight tendency for a higher rate of capsular contracture among prepectoral implants (91.1% vs. 86.9%). This might be explained by the delayed clinical detection of capsular fibrosis as a result of the overlying muscle layer as well as less exposure of the implant to breast duct bacteria, with the pectoral muscle acting as a barrier [45,46].

It is widely accepted that PMRT is one of the major risk factors for capsular contracture, yet there is not a sufficient number of radiation-related analyses on minimizing the risk. Most patients in this study (102 patients, 86.4%) were irradiated using VMAT at 50.0–50.4 Gy in 25–28 fractions, allowing for homogenous and conformal dose distributions. The maximum dose (Dmax/PTV-D2%) threshold was set to 107% of the PTV prescribed dose to further increase homogeneity. We found no significant correlation between any dosimetric characteristics (PTV-D98%, PTV-D50%, PTV-D2% and PTV-Dmean) and the occurrence of capsular contracture, presumably due to the modern radiotherapy techniques applied.

However, we observed a tendency for larger PTVs in patients developing capsular contracture. The size of the PTV is mostly defined by the implant size and the respective anatomy of the patients. A larger implant implies a larger surface area, which may also increase the risk of capsular contracture. The European Society for Radiotherapy and Oncology (ESTRO) formed a multidisciplinary initiative with breast cancer experts to define a consensus guideline for target volume delineation for PMRT following implant-based reconstruction, which aimed for anatomically risk-adapted radiation therapy planning, avoiding the inclusion of non-target tissue like the implant [47,48]. An implant-sparing approach, e.g., HALFMOON (helical altered fractionation for implant partial omission), is mainly used to reduce the radiation dose to organs at risk and optimize target coverage in patients with a subpectoral or prepectoral implant placement. However, the preliminary results on the HALFMOON concept for subpectoral implants presented at the annual ESTRO meeting in 2023 (follow-up duration: 1.2 years, available data on 32 out of 47 patients) showed a surprisingly high number of 28 out of 32 patients (87.5%) reporting ≥Baker II capsular contracture [49]. Further studies regarding the severity and incidence of capsular contracture following the proposed implant-sparing approach with longer follow-up periods are needed.

Despite advances in reconstructive procedures and radiation techniques, PMRT has mostly been applied in conventional fractionations of 25–28 fractions of 1.8–2 Gy over recent decades. Data supporting hypofractionated PMRT are still limited, especially on reconstructed breasts. Wang et al. demonstrated, in a phase 3 trial, the noninferiority of hypofractionated PMRT in terms of locoregional recurrence rates compared to conventional fractionation; however, patients with immediate breast reconstruction were excluded from the trial [50]. Several studies reported a comparable rate of severe capsular contracture for hypofractionated 3-week PMRT (15 fractions of 2.67 Gy) to those for 5-week schedules [51,52,53,54,55]. Furthermore, Kim et al. showed a significantly lower incidence of major contracture in hypofractionated PMRT [53]. Looking at the effectiveness and low toxicity profile of hypofractionated whole-breast radiotherapy, hypofractionated PMRT on reconstructed breasts may yield similar premises, yet further research is needed. Nevertheless, the ESTRO recently published a guideline stating that hypofractionated radiotherapy to the chest wall can be offered regardless of the time and type of breast reconstruction [56]. However, the current version of the NCCN guideline still recommends conventional fractionation for PMRT in the case of breast reconstruction [16].

Due to the retrospective nature of this study, the analysis is predisposed to potential selection bias. The number of patients was too small to interpret and observe the influence of different treatment characteristics. Being a time-dependent occurrence, the median follow-up of 22 months may be considered too short to observe the development of capsular contracture and other possible radiation effects. Although various dosimetric factors of the applied radiotherapy plans were analyzed, we could not detect any association between dosimetric variations and the occurrence of capsular contracture. Despite these shortcomings, this study provides an interdisciplinary analysis of a clinically relevant adverse event of PMRT on reconstructed breasts.

## 5. Conclusions

Capsular contracture is one of the major complications in breast cancer patients following immediate implant-based breast reconstruction and PMRT. Its pathogenesis is known to be complex and multifactorial. In this study, we found that postoperative complications and regional nodal metastases could further increase the risk of capsular contracture. Modern implant-sparing target volume concepts are discussed for PMRT, although these concepts have not shown reduced incidences of capsular contracture compared to conventional approaches up to now. Additional studies are needed to identify further potential risk factors which might be future targets for preventive approaches.

## Figures and Tables

**Table 1:** Patient and tumor characteristics.

Characteristics	Capsular Contracture No. (%)		Total No. (%)	*p*-Value
	Yes	No		
Patients (No., %)	56 (47.5)	62 (52.5)	118 (100.0)	
Age, years (No., %)				0.338
Age ≤ 50	39 (69.6)	48 (77.4)	87 (73.7)	
Age > 50	17 (30.4)	14 (22.6)	31 (26.3)	
BMI (Mean, SD)	24.5 (4.0)	24.6 (4.7)		0.834
Tumor stage (pT/ypT stage)				0.500
0	10 (17.9)	12 (19.4)	22 (18.6)	
is	4 (7.1)	3 (4.8)	7 (5.9)	
1	14 (25.0)	19 (30.6)	33 (28.0)	
2	17 (30.4)	23 (37.1)	40 (33.9)	
3	10 (17.9)	5 (8.1)	15 (12.7)	
4	1 (1.8)	0 (0.0)	1 (0.8)	
Nodal status (pN/ypN stage)				0.031
0	22 (39.3)	21 (33.9)	43 (36.4)	
1	22 (39.3)	37 (59.7)	59 (50.0)	
2	8 (14.3)	4 (6.5)	12 (10.2)	
3	4 (7.1)	0 (0.0)	4 (3.4)	
Menopausal status (No., %)				0.618
Premenopausal	33 (58.9)	41 (66.1)	74 (62.7)	
Perimenopausal	6 (10.7)	4 (6.5)	10 (8.5)	
Postmenopausal	17 (30.4)	17 (27.4)	34 (28.8)	
Estrogen receptor/ER (No., %)				0.247
Positive	41 (73.2)	50 (80.6)	91 (77.1)	
Negative	14 (25.0)	10 (16.1)	24 (20.3)	
Unknown	1 (1.8)	2 (3.2)	3 (2.5)	
Progesterone receptor/PR (No., %)				0.149
Positive	33 (58.9)	43 (69.4)	76 (64.4)	
Negative	23 (41.1)	17 (27.4)	40 (33.9)	
Unknown	0 (0.0)	2 (3.2)	2 (1.7)	
Her2-targeted Therapy (No., %)				0.769
Yes	13 (23.2)	13 (21.0)	26 (22.0)	
No	43 (76.8)	49 (79.0)	92 (78.0)	
Perioperative Chemotherapy (No., %)				0.153
Yes	49 (87.5)	48 (77.4)	97 (82.2)	
No	7 (12.5)	14 (22.6)	21 (17.8)	
Hormone therapy (No., %)				0.654
Yes	45 (80.4)	48 (77.4)	93 (78.8)	
No	11 (19.6)	14 (22.6)	25 (21.2)	
Surgery technique (No., %)				0.763
Nipple-sparing mastectomy	26 (46.4)	27 (43.5)	53 (44.9)	
Skin-sparing mastectomy	28 (50.0)	30 (48.4)	58 (49.2)	
Modified radical mastectomy	2 (3.6)	4 (6.5)	6 (5.1)	
Unknown	0 (0.0)	1 (1.6)	1 (0.8)	
Lymph node surgery (No., %)				0.744
Sentinel node	13 (23.2)	16 (25.8)	29 (24.6)	
Axillary dissection	43 (76.8)	46 (74.2)	89 (75.4)	
Implant position (No., %)				0.472
Subcutaneous	51 (91.1)	53 (85.5)	104 (88.1)	
Subpectoral	5 (8.9)	8 (12.9)	13 (11.0)	
Unknown	0 (0.0)	1 (1.6)		
Size, mL (median, IQR)	330.0 (255.0–445.0)	335.0 (235.0–420.0)	335.0 (238.8–437.5)	0.531
Surface (No., %)				0.622
Textured or Polyurethane	53 (94.6)	58 (93.5)	111 (94.1)	
Smooth	1 (1.8)	2 (3.2)	3 (2.5)	
Unknown	2 (3.6)	2 (3.2)	4 (3.4)	
Shape (No., %)				0.940
Round	1 (1.8)	1 (1.6)	2 (1.7)	
Anatomical	53 (94.6)	59 (95.2)	112 (94.9)	
Unknown	2 (3.6)	2 (3.2)	4 (3.4)	
Utilization of Meshes (No., %)	5 (8.9)	1 (1.6)	6 (5.1)	0.074
Post-surgery complications (No., %)				
Yes	38 (67.9)	28 (45.2)	66 (55.9)	0.017
Seroma/haematoseroma	19 (33.9)	14 (22.6)	33 (28.0)	0.187
Puncture/aspiration of seroma	7 (12.5)	8 (12.9)	15 (12.7)	0.921
Prolonged wound healing	3 (5.4)	0 (0.0)	3 (2.5)	0.065
Prolonged pain	17 (30.4)	10 (16.1)	27 (22.9)	0.073
Swelling	8 (14.3)	6 (9.7)	14 (11.9)	0.459
None	18 (32.1)	33 (53.2)	51 (43.2)	
Unknown	0 (0.0)	1 (1.6)	1 (0.8)	

**Table 2:** Radiation specification.

Radiation Specification	Capsular Contracture No. (%) or Median (IQR) or Mean (SD)		Total No. (%) or Median (IQR) or Mean (SD)	*p*-Value
	Yes	No		
Time between surgery and start of RT, weeks (Median, IQR)	9.71 (5.79–26.36)	10.36 (6.13–26.25)	10.36 (6.39–26.25)	
FB or DIBH (No., %)				0.711
FB	17 (30.4)	20 (32.2)	37 (31.4)	
DIBH	39 (69.6)	42 (67.7)	81 (68.6)	
Doses and fractionation (No., %)				0.886
50 Gy in 25 × 2 fractions	47 (83.9)	52 (83.9)	99 (83.9)	
50.4 Gy in 28 × 1.8 fractions	8 (14.3)	9 (14.5)	17 (14.4)	
Lower (termination before end of RT, 44–48 Gy)	1 (1.8)	1 (1.6)	2 (1.7)	
Tumor-bed boost (No., %)	3 (5.4)	2 (3.3)	5 (4.2)	0.580
Extent of PMRT (No, %)				0.183
Thoracic wall	4 (7.1)	7 (11.3)	11 (9.3)	
Including supra/infraclavicular	14 (25.0)	11 (17.7)	25 (21.2)	
Including supra/infraclavicular/IMA	17 (30.4)	29 (46.8)	46 (39.0)	
Including supra/infraclavicular/axillary	15 (26.8)	8 (12.9)	23 (19.5)	
Including supra/infraclavicular/ axillary/IMA	6 (10.7)	7 (11.3)	13 (11.0)	
Radiation technique (No., %)				
VMAT	49 (87.5)	53 (85.5)	102 (86.4)	
3D	7 (12.5)	9 (14.5)	16 (13.5)	
PTV thoracic wall, cm^3^ (Mean, SD)	1104.1 (402.2)	989.5 (372.3)	1036.2 (385.3)	0.264
PTV total, cm^3^ (Mean, SD)	1255.8 (382.6)	1185.9 (454.2)	1205.7 (422.2)	0.099
PTV-D98%, Gy (Mean, SD)	37.3 (8.8)	36.5 (3.9)	36.9 (6.7)	0.541
PTV-Dmean, Gy (Mean, SD)	48.7 (1.5)	49.1 (1.0)	48.9 (1.3)	0.782
PTV-D50%, Gy (Mean, SD)	49.9 (0.4)	50.1 (0.8)	50.0 (0.7)	0.737
PTV-D2%, Gy (Mean, SD)	52.7 (1.1)	53.0 (1.5)	52.8 (1.3)	0.360

FB: free breathing; DIBH: deep inspiration breath hold; IMA: internal mammary artery; VMAT: volumetric modulated arc therapy; PTV: planning target volume.

**Table 3:** Multivariate Cox regression model.

	Hazard Ratio	95% CI	*p*-Value
Pathological lymph node status			0.004
N1	1.111	0.590–2.094	0.744
N2	2.929	1.157–7.415	0.023
N3	6.739	1.979–22.949	0.002
Postoperative complications	2.245	1.204–4.187	0.011
Chemotherapy	1.714	0.701–4.192	0.238
Extent of PMRT			0.742
Chest wall inkl. supra/infra	1.688	9.525–5.421	0.379
Inkl. supra/infra/IMA	1.473	0.438–4.954	0.531
Inkl. axilla/supra/infra	2.038	0.612–6.781	0.246
Inkl. axilla/supra/infra/IMA	2.164	0.553–8.468	0.267
PTV volume, cm^3^	1.000	1.000–1.001	0.625

## Data Availability

The datasets generated for this study will not be made publicly available since national legislation and the terms of study ethics approval do not allow dataset sharing outside of the institutions participating in the analysis.
